# Considerations in planning a controlled human infection model in at-risk groups in sub-Saharan Africa: the case for pneumococcal challenge in people living with HIV in Malawi and a report of stakeholder consultation

**DOI:** 10.12688/wellcomeopenres.23277.1

**Published:** 2024-11-07

**Authors:** Klara Doherty, A Chirwa, Shalom Songolo, Alice Kusakala, E Nsomba, Pemphero Liwonde, Daniela Ferreira, Henry Mwandumba, K Jambo, S Gordon

**Affiliations:** 1 Liverpool School of Tropical Medicine Department of Clinical Sciences, Liverpool, England, UK; 2 Malawi-Liverpool Wellcome programme, Blantyre, Malawi; 3 University of Oxford Oxford Vaccine Group, Oxford, England, UK

**Keywords:** pneumococcal infections, HIV infections, vaccines, Malawi, Streptococcus pneumoniae

## Abstract

Controlled human infection models offer a unique opportunity to understand infectious disease pathogenesis and have accelerated vaccine development and evaluations in malaria and typhoid. One major limitation of most CHIMs is that they are typically conducted in healthy young adults who are generally the population least affected by infectious disease, and who exhibit distinct disease profiles to more at-risk populations such as people living with HIV, young children, and older adults. However, the added value of studying these populations with high relevance is only desirable if it can be done safely, robustly and acceptably. We present a framework to guide the conduct of a controlled human infection model in people living with HIV using a case-example of an experimental human pneumococcal carriage model in a setting of high disease-burden and transmission.

## Plain language summary

Controlled human infection models (CHIMs) are a research method in which an infection is safely introduced into volunteer participants to better in order to understand the infection and test vaccines against it. These models are more efficient than traditional clinical studies because they require fewer participants. Most CHIMs have focused on healthy young adults, who are not the ones most affected by infectious diseases and have a distinct infection profile and vaccine response compared to population with a greater burden of infectious disease such as older adults and people living with HIV. Recently researchers have started to include these high-burden populations in CHIMs but safety and ethics are critical considerations before embarking on such studies. We propose a framework for safely and ethically conducting CHIMs in people living with HIV in order to advance research in this key population. We use a case example of a CHIM of pneumococcal carriage in the nose of people living with HIV in Malawi.

## Disclaimer

The views expressed in this article are those of the author(s). Publication in Wellcome Open Research does not imply endorsement by Wellcome

## Introduction

Controlled human infection models (CHIMs) involve the deliberate introduction of an infectious agent to volunteer participants in order to study infectious disease pathogenesis or test a therapeutic intervention. In recent years CHIMs established in high-income, low endemic regions have been transferred to endemic settings to study the diseases in the populations most affected ^1– 4^. A malaria CHIM established in Oxford and Maryland has been transferred to malaria endemic settings in Kenya, Tanzania, and Gabon, to study the disease in those with prior immunity and to make the findings relevant to those most affected ^4– 6^. Similarly, an experimental human pneumococcal carriage model (EHPC) established in Liverpool has been transferred to Malawi where post-vaccine persistence of pneumococcal carriage has hindered vaccine effectiveness ^7, 8^.

CHIMs have typically been conducted in healthy, young adult volunteers. These are generally the population least affected by infectious diseases, and so efforts are being made to increase the relevance of CHIM models. For example, the UK EHPC model has been used to study pneumococcal carriage in older adults, which has indeed demonstrated a distinct infectious disease process compared to young adults ^9^. The next step for CHIMs in sub-Saharan Africa should also focus on more at-risk populations. Careful consideration of safety, public perception, and justification of the research is crucial. In Malawi, people living with HIV (PLHIV) are a key population to study pneumococcal disease and carriage and an EHPC in PLHIV in Malawi is now ongoing ^10^.

Our review of the published literature demonstrates no other CHIM in PLHIV, and no framework for CHIMs in PLHIV. Ethical frameworks and considerations for CHIMs in general have been explored, but do not address CHIMs in a potentially vulnerable population ^4, 11– 14^. CHIMs must be justified by value of the research and must present an acceptable risk both to participants and the wider community. Here we adapt the ethical frameworks developed by Binik (2020) and Miller and Grady (2001), which evaluate CHIMs in the general population, to apply them to a potentially vulnerable population such as PLHIV ^13, 14^. We explore the case for undertaking a CHIM in PLHIV using the EHPC in PLHIV in Malawi as a case-example. Additionally, we present feedback from two patient and public involvement and engagement (PPIE) workshops in Malawi in which attendees were invited to share views on CHIM in PLHIV.

## A considerations framework for controlled human infection models involving people living with HIV

Our proposed framework consists of four ethical questions, each encompassing several considerations ( Table 1).

**Table 1.** Framework for planning a controlled human infection model in an at-risk population such as PLHIV.

Ethical question	Considerations
1. Is the scientific rationale justified and does the research have translational value for the populations affected?	• Justification of the research question in the at -risk population • Adequacy of the CHIM study design • Justification of conducting the study in a low-income setting
2. Are the risks and burden of the research acceptable and can they be minimised?	• Risk and burden to individual participant • Risk and burden to community • Risk and burden to local infrastructure
3. What are the specific vulnerabilities of the target population, and should this vulnerable group be enrolled?	• Physiological vulnerability • Socio-economic vulnerability (e.g. financial hardship) • Logistical vulnerability (e.g. frequent medical appointments)
4. What is the perception from local community stakeholders and participants of controlled human infection models in the at-risk population?	• Perception of risks • Perception of benefits • Cultural factors

### 1. Is the scientific rationale for a controlled human infection model in PLHIV justified, and does the research have translational value for the populations affected?

PLHIV are a key population in addressing the burden of pneumococcal carriage and disease in settings with high HIV prevalence such as Malawi. PLHIV exhibit higher rates of pneumococcal carriage compared to HIV-uninfected and paradoxically exhibit even higher rates once established on antiretroviral therapy ^7, 15^. Pneumococcal carriage is a prerequisite for invasive pneumococcal disease (IPD) and despite the positive impact of antiretroviral therapy in reducing risk of IPD, PLHIV remain at increased risk compared to HIV-uninfected individuals ^16^. In addition, pneumococcal carriage is a precursor for transmission and PLHIV may be one of the drivers of ongoing transmission observed in Malawi following infant pneumococcal conjugate vaccine (PCV13) immunisation ^7^. Pneumococcal carriage is also a precursor for emergence of antimicrobial resistance (AMR) and PLHIV could act as a reservoir of AMR ^17^.

There is currently no vaccination policy for PLHIV in sub-Saharan Africa. The pneumococcal polysaccharide vaccine (PPV23) was harmful in PLHIV in Uganda ^18, 19^. The 7-valent pneumococcal conjugate vaccine (PCV7) was safe and effective against vaccine-serotype disease in PLHIV in Malawi, however has limited serotype coverage and no evidence for effectiveness against carriage in PLHIV ^20^. Further data on vaccine efficacy in PLHIV, immune correlates of protection from pneumococcal carriage, and dynamics of pneumococcal carriage in PLHIV are urgently required for an evidence-based vaccination strategy in sub-Saharan Africa and can be delivered by the EHPC CHIM in PLHIV.

**Adequacy of study design:** CHIMs allow researchers to control for factors which are often unknown in a community study including participant pre-exposure, microbiological and immunological status; dose, route, and strain of infectious inoculum; and environmental factors which may influence the pathogenesis of infection. Data exist on pneumococcal carriage in PLHIV, and how this differs from HIV-uninfected individuals, yet it is unclear whether this is a result of differences in immunity, or environmental and demographic factors ^21^. In addition, CHIMs offer the unique opportunity for cost-effective vaccine evaluations, which require fewer participants and a shorter study-duration than a prospective community-based study. CHIMs can efficiently up- and down-select vaccine candidates, as described on the malaria CHIM ^22^.

**Justification of study setting in a low-income country:** Conducting CHIM studies requires good clinical services, microbiology, and governance structures to allow them to be conducted safely and to acceptable standards ^12^. The World Health Organisation (WHO) released guidance highlighting the value of CHIMs for developing infrastructure and research capacity, where it meets local health priorities ^23^. The setting in Malawi is essential to answer the pertinent questions on pneumococcal carriage and vaccine efficacy in PLHIV in sub-Saharan Africa. Distinct pneumococcal vaccine efficacy has been demonstrated between Malawi and the UK ^8^. PLHIV in the UK can be expected to be different from Malawi or other low-income settings in terms of immune profile and vaccine response, as they are exposed to a higher force of infection than their UK counterparts and benefit from herd and direct immunity from PCV ^24^.

### 2. Are the risks and burden of the research acceptable and can they be minimised?

Participant safety is paramount when recruiting a potentially vulnerable group like PLHIV. This is important for study integrity, and participant and public trust in CHIMs. Binik (2020) advocates distinguishing study risks and burdens ^13^. CHIM studies may demand significant burden on participants, such as isolation and frequent sampling, even if risk of serious harm is minimal ^13^. CHIM studies must also consider risks and burdens to the local community and local infrastructure.

**Risk and burden to individual participant – **Individual risk may be evaluated by striving for a minimal risk study i.e. risk is no greater than those presented in daily life ^13^. Pneumococcal carriage is ubiquitous in PLHIV in Malawi, and thus may be considered a risk presented in daily life for this population. At any one time, 26% to 52% of PLHIV will have natural pneumococcal carriage, and 99% of PLHIV will experience at least one pneumococcal carriage event over six to ten months ^7, 15, 25^. Most of these events do not result in IPD and experimental carriage is expected to be a similar benign event ^10^. However, it is prudent to consider experimental pneumococcal inoculation more than minimal risk to ensure comprehensive safety monitoring and risk mitigation is incorporated into the study design. This can include isolating or partially isolating participants in study accommodation, regular telephone or in-person contact with participants, careful participant selection, careful inoculum selection and preparation, and comprehensive safety information instruction.

The EHPC in Malawi has recruited only immune-reconstituted and virally-suppressed PLHIV, as risk of IPD closely correlates with CD4 T-cell count ^10, 26^. Selection of a relatively benign pneumococcal serotype (6B) which rarely causes disease and employing a slow dose-escalation also mitigates risk ^27^. The EHPC model in Malawi houses inoculated participants in single-occupancy study accommodation and includes frequent telephone contact with participants between study-visits ^10^. Paradoxically, many safety provisions increase participant burden as study accommodation may take participants away from earning and family responsibilities. Evaluating the burden of the research requires input from the volunteers themselves ^14^. The EHPC in Liverpool and Malawi has been evaluated in terms of acceptability of sampling and study design to participants, but this must be an ongoing exercise for a CHIM in a vulnerable participant group ^17, 28– 30^.

**Risk and burden to community –** Risk of onward transmission exists and is particularly pertinent in PLHIV who exhibit more pneumococcal shedding than HIV-uninfected adults ^17^. Mitigation of these risks mirror those for participants and includes participant selection to exclude those in contact with vulnerable patient groups, study accommodation to limit community contact, and study clinical care extending to household contacts of participants. However, considering the high natural carriage prevalence in PLHIV, it is unlikely that experimental carriage in PLHIV provides a risk to the community above and beyond natural carriage.

**Risk and burden to local infrastructure –** Risk to local infrastructure may include burden on health-care facilities with participants attending with inoculum-associated symptoms, and burden on local economy with participants not attending work or unable to fulfil family responsibilities. The EHPC in Malawi provides a robust and experienced clinical team to manage any symptoms before the health-care system is burdened. Study follow-up visits have flexibility to allow participants to schedule visits around other commitments ^10^.

### 3. What are the specific vulnerabilities of the target population and should this vulnerable group be enrolled?

Vulnerabilities in PLHIV are physiological, socioeconomic and logistical. The EHPC in PLHIV in Malawi has been designed to provide PLHIV with population-specific information prior to participation, to account for differences in health-literacy and understanding of infection and disease ^14^. PLHIV are under regular medical care and avoiding interference with this due to frequent study visits is essential, as well as mitigating against accidental serostatus disclosure in the study design. Although serious illness because of experimental pneumococcal challenge is unlikely, PLHIV may be living with undiagnosed complication which are unveiled during study recruitment and warrant further medical assessment which may not be easily accessible.

Furthermore, PLHIV in Malawi experience more financial hardship than the HIV-uninfected population which could lead to undue influence (by remuneration) ^21^. Equally, selective non-recruitment of PLHIV because of financial hardship might be deemed unethical as financial discrimination. Financial reimbursement on the EHPC in Malawi has been determined by local guidelines on compensation, regardless of participants’ serostatus or vulnerability ^31^. Exclusion on the basis of perceived specific vulnerabilities may be considered paternalistic, and prevents PLHIV exercising their autonomy in the context of fully informed consent ^13^. Key to this process is active consultation and engagement with the community advisory groups and the local ethics committees.

### 4. What is the perception from local community stakeholders and participants of controlled human infection models in the at-risk population?

Public and participant perception is a key consideration for CHIM studies, and community consultation and stakeholder engagement prior to, and during implementation is vital to permit CHIMs to be conducted and for enhancing the scientific and ethical quality of the studies. A step-wise approach to community consultation has been described in Kenya and Malawi ^4, 28, 30^. PPIE provides a framework for CHIMs to implement safe, ethical, and acceptable studies. For example, the use of passive recruitment techniques in the EHPC in PLHIV was implemented as direct solicitation was identified by stakeholders to potentially risk unduly pressuring individuals to participate ^10, 30^. Although this approach also has potential to introduce bias, hence reducing the generalisability of the data.

The perception of CHIM in PLHIV from local community stakeholders and CHIM participants was explored in Malawi in two workshops in October 2022 and January 2023. A total of 37 attendees included PLHIV, healthcare providers, community leaders, community advisory groups, former pneumococcal CHIM participants, and ethical review board members. The perception of CHIM in PLHIV reported by attendees reflect the four ethical questions presented in our framework ( Table 1). The scientific rationale or value of the research was framed in terms of reducing the burden of respiratory illness which would “reduce much workload on [the] health sector” and in terms of generating locally relevant data to inform local vaccine programmes:

*“Why do we have to wait for results from studies done elsewhere? This is a welcome development”*

Attendees at the workshop focused on individual risk and risk mitigation, rather than community or local infrastructure risk, and focused on risk rather than burden. Attendees largely supported acceptability of CHIM in PLHIV if risk to individual participants was mitigated, for example by recruiting participants who were considered stable and well:

*“I agree that this research can be done in people who are infected with HIV but [they] must be stable”*

*“I will say two ways, yes and no. Yes, if the study will focus on PLHIV with good HIV control and no if the patient is severely sick"*

Acceptability was also contingent on mitigating risk with robust safety procedures so “if a person gets sick, they can meet [the research team] easily” and “there should be a plan so that if there is any problem, the person can get help quickly”. Attendees highlighted that these safety procedures should be clearly understood by participants:

*“My opinion is that these people should be well protected and we should give them a chance to ask questions”*

The need to maintain safety even when a participant withdraws consent was mentioned so *“if a person wants to quit they should quit safely”.* Although the risk and burden on local infrastructure and community was not directly raised by attendees, robust safety procedures necessary for individual participant safety was recognised as a mitigation against burdening local health infrastructure.

The specific vulnerabilities of PLHIV identified by attendees included differences in health literacy which would influence obtaining informed consent. The need to provide sufficient information to PLHIV participants was identified:

*“Let’s give full information so that if they want to join the study they know what it involves”*
*“Sit down with them and enlighten them thoroughly so that they understand”*

Finally, the central role of community consultation for a successful CHIM in PLHIV was repeatedly mentioned by attendees at the PPIE workshops in Malawi. Attendees highlighted the need to prevent unfounded myths and fears spreading in the community:

*“If people are not told, they will not understand and we see them as afraid because they have not been taught [...]”*

*“The study seems safe but there is need to involve the community throughout the study to clear out myths”*

The need to be sensitive to potential vaccine hesitancy was raised:

*“…it is important is that people are educated well, because we already have challenges in the communities to convince them to have some of these vaccines”*

The workshop recommended continuous community engagement prior to, and during the study to clarify myths and build a community strategy for future challenge models.

## Conclusions

The case for studying experimental pneumococcal carriage in PLHIV in Malawi is strong as there is a need to address the ongoing burden in PLHIV and the high post-vaccine residual pneumococcal carriage in Malawi. Like any human subject study, there must be a careful consideration of the potential benefits of the study, off set against the potential risks and burdens. Initial stakeholder consultation demonstrates support for CHIMs in PLHIV in Malawi as long as individual risk is mitigated against and community consultation and education is prioritised. The EHPC in PLHIV in Malawi demonstrates that this can be done safely and acceptably however ongoing community engagement and consultation is essential.

## List of abbreviations

AMR = antimicrobial resistance

CHIM = Controlled Human Infection Model

EHPC = Experimental Human Pneumococcal Carriage model

IPD = Invasive pneumococcal disease

PCV7 = 7-valent pneumococcal conjugate vaccine

PCV13 = 13-valent pneumococcal conjugate vaccine

PPV23 = pneumococcal polysaccharide vaccine

PLHIV = people living with HIV

PPIE = patient and public involvement and engagement

## Ethics and consent statement 

Ethical approval was not required to conduct the stakeholder consultation meeting. This was part of community engagement conducted by the Malawi-Liverpool Wellcome programme and the MARVELS consortium. Verbal consent was sought to engage the PPIE participants and record the discussion.

## Data Availability

No data are associated with this article
