# Supplementary material for: Considerations in planning a controlled human infection model in at-risk groups in sub-Saharan Africa: the case for pneumococcal challenge in people living with HIV in Malawi and a report of stakeholder consultation
Source: Wellcome Open Res. 2025 Nov 24;9:655. Originally published 2024 Nov 7. [Version 2] doi: 10.12688/wellcomeopenres.23277.2 (PMC12515359; doi:10.12688/wellcomeopenres.23277.2)
Supplement: Peer review history

# Reviewer report: Reviewer response for version 2

**DOI:** 10.21956/wellcomeopenres.27525.r134938

**Published:** 2025-12-11

**Authors:** Diane Gbesemete

**Affiliations:** 1 University of Southampton, Southampton, UK

The authors have adequately addressed all of my comments and have made appropriate additions to the manuscript. I have no further comments and wish them well with their ongoing studies.

Does the article adequately reference differing views and opinions?

Partly

Are all factual statements correct, and are statements and arguments made adequately supported by citations?

Yes

Is the Open Letter written in accessible language?

Yes

Where applicable, are recommendations and next steps explained clearly for others to follow?

Yes

Is the rationale for the Open Letter provided in sufficient detail?

Yes

Reviewer Expertise:

CHIMs with respiratory bacteria in the UK and Mali, public consultation / PPIE for CHIMs

I confirm that I have read this submission and believe that I have an appropriate level of expertise to confirm that it is of an acceptable scientific standard.

# Reviewer report: Reviewer response for version 2

**DOI:** 10.21956/wellcomeopenres.27525.r140543

**Published:** 2025-12-11

**Authors:** Stephen Lockhart

**Affiliations:** 1 Hurst Grange Associates, Berkshire, UK

I am satisfied that my review comments have been adequately addressed.

Does the article adequately reference differing views and opinions?

Partly

Are all factual statements correct, and are statements and arguments made adequately supported by citations?

Yes

Is the Open Letter written in accessible language?

Yes

Where applicable, are recommendations and next steps explained clearly for others to follow?

Not applicable

Is the rationale for the Open Letter provided in sufficient detail?

Partly

Reviewer Expertise:

Vaccine clinical trials and vaccine development

I confirm that I have read this submission and believe that I have an appropriate level of expertise to confirm that it is of an acceptable scientific standard.

# Reviewer report: Reviewer response for version 2

**DOI:** 10.21956/wellcomeopenres.27525.r140545

**Published:** 2025-11-25

**Authors:** James D Kellner

**Affiliations:** 1 University of Calgary, Calgary, Canada

The authors have sufficiently addressed the comments and suggestion that I made and I have no further comments. I wish them well with this project.

Does the article adequately reference differing views and opinions?

No

Are all factual statements correct, and are statements and arguments made adequately supported by citations?

Yes

Is the Open Letter written in accessible language?

Yes

Where applicable, are recommendations and next steps explained clearly for others to follow?

Yes

Is the rationale for the Open Letter provided in sufficient detail?

Yes

Reviewer Expertise:

Pediatrics, infectious diseases, vaccine preventable infections (including Streptococcus pneumoniae), vaccines, clinical epidemiology, and vaccine clinical trials.

I confirm that I have read this submission and believe that I have an appropriate level of expertise to confirm that it is of an acceptable scientific standard.

# Reviewer report: Reviewer response for version 2

**DOI:** 10.21956/wellcomeopenres.27525.r134500

**Published:** 2025-11-25

**Authors:** Andrew Gorringe

**Affiliations:** 1 UK Health Security Agency, London, UK

The authors have greatly improved the manuscript.

Does the article adequately reference differing views and opinions?

Partly

Are all factual statements correct, and are statements and arguments made adequately supported by citations?

Partly

Is the Open Letter written in accessible language?

Yes

Where applicable, are recommendations and next steps explained clearly for others to follow?

Yes

Is the rationale for the Open Letter provided in sufficient detail?

Yes

Reviewer Expertise:

Vaccine immunology (GBS, pertussis Neisseria), correlates of protection, CHIM studies

I confirm that I have read this submission and believe that I have an appropriate level of expertise to confirm that it is of an acceptable scientific standard.

# Reviewer report: Reviewer response for version 2

**DOI:** 10.21956/wellcomeopenres.27525.r140546

**Published:** 2025-11-25

**Authors:** Paul Kaye

**Affiliations:** 1 Department of Biology, Hull York Medical School (Ringgold ID: 12195), University of York, York, North Yorkshire, UK

The authors have adequately addressed the issues raised in my initial review.  I have no further comments.

Does the article adequately reference differing views and opinions?

Partly

Are all factual statements correct, and are statements and arguments made adequately supported by citations?

Yes

Is the Open Letter written in accessible language?

Yes

Where applicable, are recommendations and next steps explained clearly for others to follow?

Partly

Is the rationale for the Open Letter provided in sufficient detail?

Yes

Reviewer Expertise:

CHIM, leishmaniasis, NTDs, immunology

I confirm that I have read this submission and believe that I have an appropriate level of expertise to confirm that it is of an acceptable scientific standard.

# Reviewer report: Reviewer response for version 1

**DOI:** 10.21956/wellcomeopenres.25656.r133436

**Published:** 2025-10-11

**Authors:** Andrew Gorringe

**Affiliations:** 1 UK Health Security Agency, London, UK

This letter describes an ethical framework for conducting a CHIM in a population living with HIV in Malawi. The letter describes the various areas that should be considered in appropriate detail.

 I have had the opportunity to read the four previous reviews provided and agree with many of the points raised and see no reason to repeat these unnecessarily.

 Specific comments

 Abstract para 2 - "to better in order to" use one of these phrases. "...compared to populations with a greater..."

 Section 1. para 2 The authors should make clear that reference 20 does not assess whether colonisation is affected by PCV7. A sentence that describes the affect of PCV vaccination on pneumococcal carriage should be added with evidence from natural infection and CHIM.

 Risk and burden to the individual participant - "striving for a minimal risk study". I think "striving" is not a helpful word. What are the factors that would make a study of minimal risk for the PLHIV participants?

 Section 3 para 1 - What are "undiagnosed complications". It would be helpful if this was more specific.

 Section 4. Other reviewers have commented that the quotes from stakeholders are overwhelmingly positive, and it is difficult to get a sense of how the proposed study was received. Did any stakeholders think PLHIV individuals should not be used?

Does the article adequately reference differing views and opinions?

Partly

Are all factual statements correct, and are statements and arguments made adequately supported by citations?

Partly

Is the Open Letter written in accessible language?

Yes

Where applicable, are recommendations and next steps explained clearly for others to follow?

Yes

Is the rationale for the Open Letter provided in sufficient detail?

Yes

Reviewer Expertise:

Vaccine immunology (GBS, pertussis Neisseria), correlates of protection, CHIM studies

I confirm that I have read this submission and believe that I have an appropriate level of expertise to confirm that it is of an acceptable scientific standard.

# Response

**Published:** 2025-10-29

**Authors:** Klara Doherty

**Affiliations:** MARVELS, Malawi-Liverpool-Wellcome Trust Clinical Research Programme, Blantyre, Southern Region, Malawi

Many thanks for your review and feedback on the manuscript. We have addressed points raised by previous reviewers in version 2 of the manuscript. Your additional comments have helped strengthen the manuscript and we are grateful for your input.

 Specific comments

 Abstract para 2 - "to better in order to" use one of these phrases. "...compared to populations with a greater..."

**Response: **Many thanks for highlighting this typo. This has been corrected in version 2 of the manuscript.

 Section 1. para 2 The authors should make clear that reference 20 does not assess whether colonisation is affected by PCV7. A sentence that describes the affect of PCV vaccination on pneumococcal carriage should be added with evidence from natural infection and CHIM. **Response:** Many thanks for highlighting this error. We have corrected the manuscript to make it clear that reference 20 does not evaluate pneumococcal carriage. Excess natural pneumococcal carriage in PLHIV in the post-vaccine era is described in the earlier sections outlining the burden of disease.  

 Risk and burden to the individual participant - "striving for a minimal risk study". I think "striving" is not a helpful word. What are the factors that would make a study of minimal risk for the PLHIV participants?

**Response:** We have amended this sentence to clarify we are describing the definition of minimal risk study from reference 13. In the latter half of the paragraph we described factors that would make the study of minimal risk for participants and in the following paragraph we describe how this was done on the EHPC in Malawi.

 Section 3 para 1 - What are "undiagnosed complications". It would be helpful if this was more specific.

**Response:** Many thanks for highlighting this ambiguity. We have amended this to “PLHIV may be living with an undiagnosed condition which are unveiled during participant screening tests”

 Section 4. Other reviewers have commented that the quotes from stakeholders are overwhelmingly positive, and it is difficult to get a sense of how the proposed study was received. Did any stakeholders think PLHIV individuals should not be used?  

**Response: **Thank you for highlighting this limitation in the manuscript. We hope the revised manuscript is clearer on this point. There were stakeholders who felt PLHIV should not be used in certain contexts e.g. uncontrolled HIV, poorly adherent patients, without fully informed, population-specific consent. None of the stakeholders expressed the view that PLHIV should never be used.

# Reviewer report: Reviewer response for version 1

**DOI:** 10.21956/wellcomeopenres.25656.r133748

**Published:** 2025-10-07

**Authors:** Diane Gbesemete

**Affiliations:** 1 University of Southampton, Southampton, UK

It is increasingly recognised that controlled human infection studies can, and should, be conducted within populations of relevance where possible. However, it is imperative that such studies are designed and conducted safely, ethically, transparently, and with a high level of scientific rigour. This is especially true when the population of relevance is particularly vulnerable, whether medically or socially. This letter provides a framework of ethical questions used to facilitate consideration of such a study, using the example of a pneumococcal CHIM in people living with HIV in Malawi. The scientific and ethical justifications for this study are discussed, with evidence of appropriate public consultation and stakeholder agreement. The framework provided can be adapted to facilitate the consideration of future CHIMs in other vulnerable populations, both in LMICs and HICs. The letter is therefore very relevant to the current direction of CHIM research and is well-written, I have just a few comments.

1. Scientific rationale - justification of the research question – the case for using CHIMs within vulnerable populations in general is explained well throughout the manuscript, and the importance of investigating pneumococcal carriage in this specific population is also explained well. However, given that natural pneumococcal carriage is reported to be so prevalent among PLHIV in Malawi, it is not fully explained why a CHIM is necessary to answer the pertinent questions in this specific population, in comparison to epidemiological studies or field trials. I have therefore answered "partly" to Q2.
2. Adequacy of study design – this section repeats much of the argument regarding the importance of CHIMs and of investigating pneumococcal colonisation in PLHIV but does not discuss specific aspects of study design. While a full summary of a specific protocol is outside of the remit of this manuscript, some specific points demonstrating how the study design would ensure participant safety, data integrity and maximal scientific and social value would be useful in this section.
3. Risks and burdens – the discussion around potential risks and burdens is comprehensive and it is good that many non-medical risks have been considered as well as the more obvious medical ones. When considering clinical safety, it is clearly mentioned that participants will be closely monitored for occurrence of pneumococcal disease, but it is not explicitly mentioned how any concerns around possible disease would be assessed or managed, i.e. with antibiotic clearance or rescue therapy.
4. Community consultation – a large proportion of the manuscript describes the public consultation and quotes from stakeholders which highlights the importance of such consultation. The section is arranged to match the order of the framework which helps to reference different viewpoints back to the framework, although it this could be made more obvious e.g. with subheadings for the different areas of discussion. It is notable that the public views expressed are all very positive and in support of the study going ahead. Were there any negative viewpoints or concerns around risks or burdens expressed?

Does the article adequately reference differing views and opinions?

Partly

Are all factual statements correct, and are statements and arguments made adequately supported by citations?

Yes

Is the Open Letter written in accessible language?

Yes

Where applicable, are recommendations and next steps explained clearly for others to follow?

Yes

Is the rationale for the Open Letter provided in sufficient detail?

Yes

Reviewer Expertise:

CHIMs with respiratory bacteria in the UK and Mali, public consultation / PPIE for CHIMs

I confirm that I have read this submission and believe that I have an appropriate level of expertise to confirm that it is of an acceptable scientific standard, however I have significant reservations, as outlined above.

# Response

**Published:** 2025-10-29

**Authors:** Klara Doherty

**Affiliations:** MARVELS, Malawi-Liverpool-Wellcome Trust Clinical Research Programme, Blantyre, Southern Region, Malawi

Many thanks for your careful review of the manuscript. We are very grateful for your suggestions and feel they have helped us strengthen the manuscript. We hope the revised manuscript addresses your reservations.

- Scientific rationale - justification of the research question – the case for using CHIMs within vulnerable populations in general is explained well throughout the manuscript, and the importance of investigating pneumococcal carriage in this specific population is also explained well. However, given that natural pneumococcal carriage is reported to be so prevalent among PLHIV in Malawi, it is not fully explained why a CHIM is necessary to answer the pertinent questions in this specific population, in comparison to epidemiological studies or field trials. I have therefore answered "partly" to Q2.

**Response:** Many thanks for highlighting this important point. We have revised the manuscripts to include a more detailed discussion on the limitations of the EHPC and the alternative approaches such as community field trials which carry the key limitation of unclear timing of pneumococcal exposure.

- Adequacy of study design – this section repeats much of the argument regarding the importance of CHIMs and of investigating pneumococcal colonisation in PLHIV but does not discuss specific aspects of study design. While a full summary of a specific protocol is outside of the remit of this manuscript, some specific points demonstrating how the study design would ensure participant safety, data integrity and maximal scientific and social value would be useful in this section.

**Response: **Version 2 of the manuscript includes more detail on management of adverse events and participant selection criteria to ensure participant safety on the EHPC. We have also included a more explicit description of how data from the EHPC could help inform vaccine policy for maximal scientific and social value.

- Risks and burdens – the discussion around potential risks and burdens is comprehensive and it is good that many non-medical risks have been considered as well as the more obvious medical ones. When considering clinical safety, it is clearly mentioned that participants will be closely monitored for occurrence of pneumococcal disease, but it is not explicitly mentioned how any concerns around possible disease would be assessed or managed, i.e. with antibiotic clearance or rescue therapy.

**Response: **We appreciate this request for detail. The revised manuscript includes the detail that participants are pro-actively managed by the clinical study team to prevent burdening of local health service. Assessment and management of any disease are thus determined by the clinician. More detail of the EHPC in Malawi is specified in the published study protocol and includes predefined agreements with a public and private hospital in Blantyre, Malawi. This manuscript serves to highlight ethical considerations, but precise operational details must be study and site specific.

- Community consultation – a large proportion of the manuscript describes the public consultation and quotes from stakeholders which highlights the importance of such consultation. The section is arranged to match the order of the framework which helps to reference different viewpoints back to the framework, although it this could be made more obvious e.g. with subheadings for the different areas of discussion. It is notable that the public views expressed are all very positive and in support of the study going ahead. Were there any negative viewpoints or concerns around risks or burdens expressed?

**Response:** Thank you for the suggestions. We have added subheadings to section 4 to make the structure clearer. The text has also been amended to make it clearer that some participants did express hesitation with CHIMs in PLHIV but only in certain contexts e.g. in uncontrolled HIV, with poorly adherent patients, without scientific data to justify the research. This has been made clearer in version 2 of the manuscript.

# Reviewer report: Reviewer response for version 1

**DOI:** 10.21956/wellcomeopenres.25656.r128819

**Published:** 2025-09-27

**Authors:** Stephen Lockhart

**Affiliations:** 1 Hurst Grange Associates, Berkshire, UK

This letter presents a proposed framework of things to consider when planning a controlled human infection model (CHIM) in at-risk groups in sub-Saharan Africa.  As an exemplar it describes the case for a pneumococcal controlled human infection model (CHIM) in people living with HIV (PLHIV) in Malawi, including presentation of outputs from stakeholder meetings.  

 The authors are very experienced in this field, with a prior pneumococcal conjugate vaccine (PCV) efficacy trial in Malawi showing efficacy against invasive pneumococcal disease (IPD) in a population largely PLHIV, and an established CHIM for vaccine efficacy in pneumococcal carriage in healthy adults in UK and Malawi.

 The considerations are based upon two sets of ethical frameworks for CHIM studies, although I am unable to review these as they are both behind paywalls.  Nonetheless the list of considerations in Table 1 appears reasonable.  Most of this paper is entirely correct and reasonable so I am just commenting where there might be room for improvement.

**1. Is the scientific rationale for a controlled human infection model in PLHIV justified, and does the research have translational value for the populations affected?**

 It is stated that there is no evidence of PCV7 preventing carriage based on reference 20, but for clarity that trial simply did not examine carriage; there is absence of evidence not evidence of absence.  Most importantly that trial showed efficacy against vaccine type IPD, which in my opinion would do more to justify use of PCVs in PLHIV (now with more extended serotype coverage) than a CHIM carriage efficacy study. There is evidence of PCV10-HiD reducing carriage in HIV infected/exposed infants in South Africa. 

 (Madhi 2020 https://doi.org/10.1016/j.vaccine.2020.01.062)

 However, I am not aware of a trial of PCV in prevention of natural carriage in adult PLHIV.  Such a trial might fill a gap in information supporting policy discussions, but I agree that it would be more complex, take longer and probably be more expensive than a CHIM efficacy study.

 It is stated that “Further data on vaccine efficacy in PLHIV, immune correlates of protection from pneumococcal carriage, and dynamics of pneumococcal carriage in PLHIV are urgently required for an evidence-based vaccination strategy in sub-Saharan Africa and can be delivered by the EHPC CHIM in PLHIV”.  It would be very useful to describe how the CHIM data would contribute to policy decisions.

**Adequacy of study design.**

 The study design is not presented, though I assume it would be similar to the study design of existing pneumococcal carriage CHIM studies. This might be a useful place to describe selection criteria which would be important in ensuring participant safety. It would also be of interest to understand a bit more about the reasons for selection of a challenge strain.

**Justification of study setting in a low-income country.**

  It is stated that “Distinct pneumococcal vaccine efficacy has been demonstrated between Malawi and the UK”. I assume that this means distinct differences.  In the reference quoted CHIM efficacy in Malawi was 62%, (95% CI 28–80) compared to 78%, (95% CI 48–91), which is not remarkable given that these were in two separate populations and CIs are widely overlapping.  Nonetheless, the evidence for a high force of infection does suggest that people in Malawi may experience more frequent exposure leading to a different immunological profile

**2. Are the risks and burden of the research acceptable and can they be minimised?**

 All good, no comments

**3. What are the specific vulnerabilities of the target population, and should this** vulnerable group be enrolled?

 All good, no comments

**4. What is the perception from local community stakeholders and participants of controlled human infection models in the at-risk population?**

 The stakeholder meetings are important and there appears to be much useful feedback.  Quantitative analysis is not really feasible, and it is reasonable to present results in the format used here.  Use of anecdotal statements is subject to bias so it might be useful to have more information on who led the meeting discussions and who selected quotations from the outputs, or at least a comment on potential biases in reporting.

Does the article adequately reference differing views and opinions?

Partly

Are all factual statements correct, and are statements and arguments made adequately supported by citations?

Yes

Is the Open Letter written in accessible language?

Yes

Where applicable, are recommendations and next steps explained clearly for others to follow?

Not applicable

Is the rationale for the Open Letter provided in sufficient detail?

Partly

Reviewer Expertise:

Vaccine clinical trials and vaccine development

I confirm that I have read this submission and believe that I have an appropriate level of expertise to confirm that it is of an acceptable scientific standard, however I have significant reservations, as outlined above.

# Response

**Published:** 2025-10-06

**Authors:** Klara Doherty

**Affiliations:** MARVELS, Malawi-Liverpool-Wellcome Trust Clinical Research Programme, Blantyre, Southern Region, Malawi

***We thank Reviewer 4 for their supportive review and detailed comments. We have made a number of corrections and clarifications which we hope improves the quality of the manuscript. We have acknowledged some additional limitations which the reviewer highlighted.*** This letter presents a proposed framework of things to consider when planning a controlled human infection model (CHIM) in at-risk groups in sub-Saharan Africa.  As an exemplar it describes the case for a pneumococcal controlled human infection model (CHIM) in people living with HIV (PLHIV) in Malawi, including presentation of outputs from stakeholder meetings.  

 The authors are very experienced in this field, with a prior pneumococcal conjugate vaccine (PCV) efficacy trial in Malawi showing efficacy against invasive pneumococcal disease (IPD) in a population largely PLHIV, and an established CHIM for vaccine efficacy in pneumococcal carriage in healthy adults in UK and Malawi.

 The considerations are based upon two sets of ethical frameworks for CHIM studies, although I am unable to review these as they are both behind paywalls.  Nonetheless the list of considerations in Table 1 appears reasonable.  Most of this paper is entirely correct and reasonable so I am just commenting where there might be room for improvement.

**1. Is the scientific rationale for a controlled human infection model in PLHIV justified, and does the research have translational value for the populations affected?**

 It is stated that there is no evidence of PCV7 preventing carriage based on reference 20, but for clarity that trial simply did not examine carriage; there is absence of evidence not evidence of absence.  Most importantly that trial showed efficacy against vaccine type IPD, which in my opinion would do more to justify use of PCVs in PLHIV (now with more extended serotype coverage) than a CHIM carriage efficacy study. There is evidence of PCV10-HiD reducing carriage in HIV infected/exposed infants in South Africa. 

 (Madhi 2020 https://doi.org/10.1016/j.vaccine.2020.01.062)

 However, I am not aware of a trial of PCV in prevention of natural carriage in adult PLHIV.  Such a trial might fill a gap in information supporting policy discussions, but I agree that it would be more complex, take longer and probably be more expensive than a CHIM efficacy study. It is stated that “Further data on vaccine efficacy in PLHIV, immune correlates of protection from pneumococcal carriage, and dynamics of pneumococcal carriage in PLHIV are urgently required for an evidence-based vaccination strategy in sub-Saharan Africa and can be delivered by the EHPC CHIM in PLHIV”.  It would be very useful to describe how the CHIM data would contribute to policy decisions.

- ***Response: Thank you for these helpful comments. We have corrected the statement regarding PCV7 (reference 20) to clarify that the trial did not assess carriage. We also expanded the rationale to explain how CHIM data could inform policy — specifically by providing data on vaccine efficacy against carriage to evaluate potential for herd immunity and by identifying immune correlates of protection to accelerate vaccine evaluations and licensure of vaccine candidates.***

**Adequacy of study design.**

 The study design is not presented, though I assume it would be similar to the study design of existing pneumococcal carriage CHIM studies. This might be a useful place to describe selection criteria which would be important in ensuring participant safety. It would also be of interest to understand a bit more about the reasons for selection of a challenge strain.

- ***Response: We have added detail under “Adequacy of study design” and “Risk and burden…” to describe participant selection and limitations of using a single serotype. The full study design and selection criteria are detailed in the published study protocol.***

 
**Justification of study setting in a low-income country.**

  It is stated that “Distinct pneumococcal vaccine efficacy has been demonstrated between Malawi and the UK”. I assume that this means distinct differences.  In the reference quoted CHIM efficacy in Malawi was 62%, (95% CI 28–80) compared to 78%, (95% CI 48–91), which is not remarkable given that these were in two separate populations and CIs are widely overlapping.  Nonetheless, the evidence for a high force of infection does suggest that people in Malawi may experience more frequent exposure leading to a different immunological profile

- ***Response: We appreciate the observation. We have revised the manuscript to clarify that the point estimates were lower in Malawi (consistent with epidemiological observations of ongoing post-vaccine transmission in Malawi) although confidence intervals overlap.***

**2. Are the risks and burden of the research acceptable and can they be minimised?**

 All good, no comments

**3. What are the specific vulnerabilities of the target population, and should this** **vulnerable group be enrolled?**

 All good, no comments

**4. What is the perception from local community stakeholders and participants of controlled human infection models in the at-risk population?**

 The stakeholder meetings are important and there appears to be much useful feedback.  Quantitative analysis is not really feasible, and it is reasonable to present results in the format used here.  Use of anecdotal statements is subject to bias so it might be useful to have more information on who led the meeting discussions and who selected quotations from the outputs, or at least a comment on potential biases in reporting.

- ***Response: We agree that potential reporting biases should be acknowledged. We have added a statement to Section 4 noting that while meetings were participant-led, outputs may be liable to bias.***

# Reviewer report: Reviewer response for version 1

**DOI:** 10.21956/wellcomeopenres.25656.r133435

**Published:** 2025-09-26

**Authors:** Paul Kaye

**Affiliations:** 1 Department of Biology, Hull York Medical School (Ringgold ID: 12195), University of York, York, North Yorkshire, UK

As the breadth of CHIM studies expands, notably to LMIC settings, this is a timely case study-based framework for inclusion of participants from vulnerable groups (in this case PLHIV).  The article is reasonable well written, nicely structured and easily accessible to experts and those entering the field of CHIM research.  It covers some new ground and is a helpful addition to the literature.  However, whilst posing many questions, the paper does not always provide the answers. For example, differences in the financial circumstances of PLHIV are noted, potentially at odds with the local recommendation for a standard reimbursement rate irrespective of vulnerabilities (Ref 31).  How was this difference in views reconciled?  The manuscript also suffers a little from poor editing, with numerous grammatical errors. These should be corrected for final indexing.

 I have a few minor comments that the authors could also consider:

**Introduction** – was the “feedback” from PPIE sessions formalised through a qualitative research study design or just informal? Please specify.

**Table 1**

 Q 1

 Ethical– does “population affected” refer only to participants or also to the community, given PLHIV may be highly infectious? Please clarify and expand statement as necessary

 Considerations – perhaps an additional point is “what alternate strategies to answer the question have been considered”?

  Q2

 Considerations – maybe a little off base, but "reputational risk to CHIM research" could also be considered here

**Risk and burden to individual participants**

- para 2 of this section indicates participants are “housed … in single-occupancy study accommodation”. This might be read as rather punitive without some further indication of duration of confinement, facilities available etc
- para 2 also mentions the benign nature of the 6B strain.  To what extent does this undermine some of the objectives of the study? A comment on how well vaccine efficacy or ICP defined using 6B translate to more virulent strains causing IPD would be helpful.

Does the article adequately reference differing views and opinions?

Partly

Are all factual statements correct, and are statements and arguments made adequately supported by citations?

Yes

Is the Open Letter written in accessible language?

Yes

Where applicable, are recommendations and next steps explained clearly for others to follow?

Partly

Is the rationale for the Open Letter provided in sufficient detail?

Yes

Reviewer Expertise:

CHIM, leishmaniasis, NTDs, immunology

I confirm that I have read this submission and believe that I have an appropriate level of expertise to confirm that it is of an acceptable scientific standard, however I have significant reservations, as outlined above.

# Response

**Published:** 2025-10-06

**Authors:** Klara Doherty

**Affiliations:** MARVELS, Malawi-Liverpool-Wellcome Trust Clinical Research Programme, Blantyre, Southern Region, Malawi

***We thank Reviewer 3 for their positive assessment and useful suggestions. We have included many of the suggestions which improved the quality of the manuscript, and we hope we have improved the clarity of the manuscript.*** As the breadth of CHIM studies expands, notably to LMIC settings, this is a timely case study-based framework for inclusion of participants from vulnerable groups (in this case PLHIV).  The article is reasonable well written, nicely structured and easily accessible to experts and those entering the field of CHIM research.  It covers some new ground and is a helpful addition to the literature.  However, whilst posing many questions, the paper does not always provide the answers. For example, differences in the financial circumstances of PLHIV are noted, potentially at odds with the local recommendation for a standard reimbursement rate irrespective of vulnerabilities (Ref 31).  How was this difference in views reconciled?  The manuscript also suffers a little from poor editing, with numerous grammatical errors. These should be corrected for final indexing.

  

- ***Response: We thank the reviewer for raising this important point. We have amended the final sentence of the section on financial reimbursement to make clear that resolution of these conflicts relies on ongoing consultation with community advisory groups, ethics committees, and participants. Our intention is not to completely resolve inevitable ethical tensions inherent in conducting CHIMs, but to highlight areas requiring careful consideration and give an example of how it was addressed in the Malawi EHPC. The important step in ethical considerations is to identify, consider, and discuss these issues. We have also undertaken a careful edit of the manuscript to address grammatical issues prior to indexing,***

  I have a few minor comments that the authors could also consider:

**Introduction** – was the “feedback” from PPIE sessions formalised through a qualitative research study design or just informal? Please specify.

- ***Response: We have clarified in Section 4 that meetings followed an unstructured format but were audio-recorded, transcribed, and analysed using thematic content analysis.***

**Table 1**

 Q 1

 Ethical– does “population affected” refer only to participants or also to the community, given PLHIV may be highly infectious? Please clarify and expand statement as necessary

- ***Response: Thank you. This question has been amended to: “1. Is the scientific rationale justified and does the research have translational value for the populations affected by the disease?”***

Considerations – perhaps an additional point is “what alternate strategies to answer the question have been considered”?

- ***Response: Thank you for the suggestion. This is a very useful additional point to consider and we have amended table 1 accordingly. We have also included a discussion of alternative strategies and limitations of CHIMs under “Adequacy of study design” subheading.***

Q2

 Considerations – maybe a little off base, but "reputational risk to CHIM research" could also be considered here

- ***Response: Thank for this suggestion. We agree that reputational risk is a critical factor, particularly in Malawi and other similar contexts. We have made this more explicit in Section 4 by noting that stakeholder consultation is essential to preserve the reputation of CHIM research and maintain trust.***

**Risk and burden to individual participants**

- para 2 of this section indicates participants are “housed … in single-occupancy study accommodation”. This might be read as rather punitive without some further indication of duration of confinement, facilities available etc
- para 2 also mentions the benign nature of the 6B strain.  To what extent does this undermine some of the objectives of the study? A comment on how well vaccine efficacy or ICP defined using 6B translate to more virulent strains causing IPD would be helpful.
- ***Response: Thank you for these comments. We have clarified that participants are housed for three days post-inoculation in study accommodation. We also expanded the discussion of limitations of using a single benign serotype (6B), while noting that immune mechanisms are largely conserved across serotypes.***

# Reviewer report: Reviewer response for version 1

**DOI:** 10.21956/wellcomeopenres.25656.r128816

**Published:** 2025-09-05

**Authors:** Emmanuella Driciru

**Affiliations:** 1 Vaccines theme, MRC/UVRI and LSHTM Uganda Research Unit (Ringgold ID: 47968), Entebbe, Uganda; 2 Uganda Virus Research Institute, Entebbe, Uganda

This manuscript clearly articulates a wide range of critical considerations in regard to CHIMs, with sufficient attention to vulnerability of the target population in question, going further to tackle financial reimbursement considerations unique to the African LMIC setting.  However, I have got just a few comments (and a question out of curiosity) for the authors.

1. The author does mention considerations that underscore such a study, safety being one of these. However, considering that the target population is one that is immunologically vulnerable (immune reconstitution based on CD4+ is plausible and the “gold standard” but does not equate to a fully competent immune repertoire thus participants still carry an immune disadvantage over the existing “healthy adult subjects” participant selection criteria for CHI studies). More so this being one of the first studies in a vulnerable population in this setting, the author surely ought to look into the immunological considerations in the article as well, to ensure a wholistic approach for a safe conduct of this model. “Frame 1” does mention physiological vulnerability in the article in regard to this and the author does give a brief section on HIV, ART and pneumococcal carriage. However, for a first time study in a vulnerable, again a more in depth discussion encompassing aspects of the physiological, immunological risks, interplay and potential AEs in the context of HIV and ART will likely be met, either potential/expected  and or from previous literature.
2. The response "partly" to question two above: “Pneumococcal carriage is ubiquitous in PLHIV in Malawi” points to alternative study designs and approaches could be used to answer the same research questions this CHI model wishes to address, that do not require the additional of risk of experimental pneumococcal carriage exposure of such a vulnerable group. Thus, discussing these in the manuscript as well would provide a more balanced discussion of the alternatives versus the preferred design (CHI model) presented here.
3. The response "partly" to question five above: measures to minimize risk of IPD such as strain of challenge agent are well discussed, however, despite stating the unlikely event of IPD occurring due to the preventive measures in place, I was expecting specific attention given to how IPD will be managed beyond the general mention of “robust and experienced clinical team to manage any symptoms before health-care system is burdened” in the manuscript. What extend of symptoms are/will be managed by the team and which ones will be referred to a hospital/medical facility outside the research clinical facility conducting the study?
4. In the first paragraph of the section titled “Risk and burden to individual participants”, the author presents two of the approaches for participant monitoring as “mutually exclusive” methods in the study design in the sense that one can replace the other. Yet regular telephone calls does not replace in-person contact and vice versa. The two approaches rather complement each other.
5. Out of curiosity regarding community perspective, among the PLHIV interviewed, was there a direct expression of willingness to participate in the study besides the views captured in this manuscript that highlight a general acknowledgement of the significance of implementing this CHI model among vulnerable populations. What was their response to a question along the lines of “would you consider participating/ be willing to participate in this study if you fit eligibility criteria?” (just in case such a question was posed to them during the time they were interviewed).

Does the article adequately reference differing views and opinions?

Partly

Are all factual statements correct, and are statements and arguments made adequately supported by citations?

Yes

Is the Open Letter written in accessible language?

Yes

Where applicable, are recommendations and next steps explained clearly for others to follow?

Partly

Is the rationale for the Open Letter provided in sufficient detail?

Yes

Reviewer Expertise:

Immunology, NTDs, Schistosomiasis CHIMs and challenge agent production, molecular biology and clinical microbiology

I confirm that I have read this submission and believe that I have an appropriate level of expertise to confirm that it is of an acceptable scientific standard, however I have significant reservations, as outlined above.

# Response

**Published:** 2025-10-06

**Authors:** Klara Doherty

**Affiliations:** MARVELS, Malawi-Liverpool-Wellcome Trust Clinical Research Programme, Blantyre, Southern Region, Malawi

***We are grateful to reviewer 2 for the thoughtful feedback. In response, we have expanded the discussion of the limitations of CHIMs and the alternatives to a CHIM and have improved the clarity of the manuscript in a number of sections.*** This manuscript clearly articulates a wide range of critical considerations in regard to CHIMs, with sufficient attention to vulnerability of the target population in question, going further to tackle financial reimbursement considerations unique to the African LMIC setting.  However, I have got just a few comments (and a question out of curiosity) for the authors.

1. The author does mention considerations that underscore such a study, safety being one of these. However, considering that the target population is one that is immunologically vulnerable (immune reconstitution based on CD4+ is plausible and the “gold standard” but does not equate to a fully competent immune repertoire thus participants still carry an immune disadvantage over the existing “healthy adult subjects” participant selection criteria for CHI studies). More so this being one of the first studies in a vulnerable population in this setting, the author surely ought to look into the immunological considerations in the article as well, to ensure a wholistic approach for a safe conduct of this model. “Frame 1” does mention physiological vulnerability in the article in regard to this and the author does give a brief section on HIV, ART and pneumococcal carriage. However, for a first time study in a vulnerable, again a more in depth discussion encompassing aspects of the physiological, immunological risks, interplay and potential AEs in the context of HIV and ART will likely be met, either potential/expected  and or from previous literature.
  - ***Response: This is a very important point. CD4 count alone does not fully capture immune reconstitution. Indeed, an objective of the EHPC in PLHIV is to explore the complex immune mechanisms underlying high pneumococcal carriage in this population and how this differs from HIV-uninfected adults. While this Open Letter focuses on ethical considerations rather than detailed inclusion/exclusion criteria, we now clarify that CD4 count was only one of several clinical criteria. The published study protocol (referenced) includes more detail on inclusion/exclusion criteria for example the exclusion of PLHIV with recent medical attendances or prior pneumococcal disease who despite a ‘good’ CD4 count may still be immunologically vulnerable. We have revised the manuscript to reflect this.***
2. The response "partly" to question two above: “Pneumococcal carriage is ubiquitous in PLHIV in Malawi” points to alternative study designs and approaches could be used to answer the same research questions this CHI model wishes to address, that do not require the additional of risk of experimental pneumococcal carriage exposure of such a vulnerable group. Thus, discussing these in the manuscript as well would provide a more balanced discussion of the alternatives versus the preferred design (CHI model) presented here.
  - ***Response: We agree that it is important to acknowledge alternative study designs. We have expanded the section “Adequacy of study design” to include a discussion of CHIM limitations (e.g. single-serotype challenge) and to note that community trials could be considered, while outlining challenges in defining timing of exposure. We believe this helps place the CHIM model in a balanced context.***
3. The response "partly" to question five above: measures to minimize risk of IPD such as strain of challenge agent are well discussed, however, despite stating the unlikely event of IPD occurring due to the preventive measures in place, I was expecting specific attention given to how IPD will be managed beyond the general mention of “robust and experienced clinical team to manage any symptoms before health-care system is burdened” in the manuscript. What extend of symptoms are/will be managed by the team and which ones will be referred to a hospital/medical facility outside the research clinical facility conducting the study?
  - ***Response: We appreciate this request for detail. While the full management plan is specified in the EHPC study protocol, we have clarified in the manuscript that all adverse events were managed by the clinical team on site, with predefined agreements for referral to partner hospitals in Blantyre if needed. We emphasise that the manuscript’s purpose is to highlight the ethical requirement to minimise burden on local health infrastructure, while the precise operational details are study specific***
4. In the first paragraph of the section titled “Risk and burden to individual participants”, the author presents two of the approaches for participant monitoring as “mutually exclusive” methods in the study design in the sense that one can replace the other. Yet regular telephone calls does not replace in-person contact and vice versa. The two approaches rather complement each other.
  - ***Response: We agree that telephone and in-person follow-up are complementary rather than mutually exclusive. The wording has been corrected accordingly.***
5. Out of curiosity regarding community perspective, among the PLHIV interviewed, was there a direct expression of willingness to participate in the study besides the views captured in this manuscript that highlight a general acknowledgement of the significance of implementing this CHI model among vulnerable populations. What was their response to a question along the lines of “would you consider participating/ be willing to participate in this study if you fit eligibility criteria?” (just in case such a question was posed to them during the time they were interviewed).
  - ***Response: We agree this would have been a valuable addition. Unfortunately, willingness to personally participate was not directly asked in the workshops.***

# Reviewer report: Reviewer response for version 1

**DOI:** 10.21956/wellcomeopenres.25656.r116886

**Published:** 2025-02-07

**Authors:** James D Kellner

**Affiliations:** 1 University of Calgary, Calgary, Canada

This open letter is an interesting and well-conceived manuscript including recognized experts on the topic of controlled human infection models (CHIMs) in at-risk groups in Africa. I have just a few comments to offer, in response to the Peer Review Form questions and a careful reading of the article.

1. I responded “No” to the question about adequately referencing differing views and opinions. While the authors do present data from their two focus group workshops on perceptions about CHIMs, including risks and benefits, they do not present any information on the scientific limitations and alternative approaches to CHIMs to measure infectious disease pathogenesis and to evaluate vaccines in their population of interest.
2. The manuscript includes several statements made attendees at the focus group workshops regarding perceptions about CHIMs. However, the authors do not mention whether they conducted formal qualitative research methods and analysis, and whether the included statements reflect formal thematic analysis or informal review of the information obtained at the workshops. Either approach is acceptable, but it should be clarified.
3. In the discussion of risk and burden to individual participants and to the community, the authors justify their pneumococcal carriage model on the basis of a low risk of invasive pneumococcal disease (IPD) occurring after experimental carriage with a “relatively benign pneumococcal serotype (6B)” and on the basis of only including persons living with HIV (PLHIV) with a sufficiently high CD4 T-cell count. However, the authors will know that non-invasive pneumonia is by far the most common serious disease that occurs after pneumococcal carriage, and it is far more common than IPD. Yet there is no mention of pneumococcal pneumonia in the manuscript, either to estimate the incidence, or to acknowledge that this is the most important serious disease to consider.
4. Related to 3) above, there is no mention of whether precautions should be taken, at the time of inoculation with pneumococcus, in persons with acute respiratory tract infections, which may be caused by viruses e.g., influenza, respiratory syncytial virus, that may antecedent infections before secondary pneumococcal pneumonia. I expect these precautions are taken but they should be mentioned in the list of mitigations considered for the risk and burden to individuals and the community.

Does the article adequately reference differing views and opinions?

No

Are all factual statements correct, and are statements and arguments made adequately supported by citations?

Yes

Is the Open Letter written in accessible language?

Yes

Where applicable, are recommendations and next steps explained clearly for others to follow?

Yes

Is the rationale for the Open Letter provided in sufficient detail?

Yes

Reviewer Expertise:

Pediatrics, infectious diseases, vaccine preventable infections (including Streptococcus pneumoniae), vaccines, clinical epidemiology, and vaccine clinical trials.

I confirm that I have read this submission and believe that I have an appropriate level of expertise to confirm that it is of an acceptable scientific standard, however I have significant reservations, as outlined above.

# Response

**Published:** 2025-10-06

**Authors:** Klara Doherty

**Affiliations:** MARVELS, Malawi-Liverpool-Wellcome Trust Clinical Research Programme, Blantyre, Southern Region, Malawi

***We thank reviewer 1 for the constructive comments which have helped strengthen the manuscript. In particular, we have expanded the discussion of the limitations of CHIMs and the alternatives to a CHIM and have improved the clarity of the manuscript in a number of sections.*** This open letter is an interesting and well-conceived manuscript including recognized experts on the topic of controlled human infection models (CHIMs) in at-risk groups in Africa. I have just a few comments to offer, in response to the Peer Review Form questions and a careful reading of the article.

1. I responded “No” to the question about adequately referencing differing views and opinions. While the authors do present data from their two focus group workshops on perceptions about CHIMs, including risks and benefits, they do not present any information on the scientific limitations and alternative approaches to CHIMs to measure infectious disease pathogenesis and to evaluate vaccines in their population of interest.
  - ***Response: Thank you for this observation. We agree that the manuscript should more explicitly acknowledge scientific limitations and alternatives. We have added a discussion under “Adequacy of study design” describing the limitation of using a single-serotype model and noting that community trials may also be feasible given the ubiquity of pneumococcal carriage. Under “Risks and burden to individual participants,” we now also discuss representativeness of CHIM participants. We hope this addition provides a more balanced perspective. ***
2. The manuscript includes several statements made attendees at the focus group workshops regarding perceptions about CHIMs. However, the authors do not mention whether they conducted formal qualitative research methods and analysis, and whether the included statements reflect formal thematic analysis or informal review of the information obtained at the workshops. Either approach is acceptable, but it should be clarified.
  - ***Response: Thank you for highlighting this need for clarity. We have revised Section 4 (“What is the perception…”) to explicitly state that while the focus group workshops were not formal qualitative studies, we employed structured elements (recording, transcription, and thematic review) to analyse participant perspectives. ***
3. In the discussion of risk and burden to individual participants and to the community, the authors justify their pneumococcal carriage model on the basis of a low risk of invasive pneumococcal disease (IPD) occurring after experimental carriage with a “relatively benign pneumococcal serotype (6B)” and on the basis of only including persons living with HIV (PLHIV) with a sufficiently high CD4 T-cell count. However, the authors will know that non-invasive pneumonia is by far the most common serious disease that occurs after pneumococcal carriage, and it is far more common than IPD. Yet there is no mention of pneumococcal pneumonia in the manuscript, either to estimate the incidence, or to acknowledge that this is the most important serious disease to consider.
  - ***Response: We agree that non-invasive pneumonia is the most common serious manifestation following carriage and that this required clearer discussion. We have amended the text to use the broader term “pneumococcal disease” rather than “IPD” as the associated reference does demonstrate CD4 count associated with pneumonia. We have also added a sentence under the section “Is the scientific rationale… justified?” to emphasise the importance of pneumonia in PLHIV. ***
4. Related to 3) above, there is no mention of whether precautions should be taken, at the time of inoculation with pneumococcus, in persons with acute respiratory tract infections, which may be caused by viruses e.g., influenza, respiratory syncytial virus, that may antecedent infections before secondary pneumococcal pneumonia. I expect these precautions are taken but they should be mentioned in the list of mitigations considered for the risk and burden to individuals and the community.
  - ***Response: Participants with any symptom of upper respiratory tract infection were screened out of the study. We agree this precaution should be explicit. We have amended the section “Risk and burden to individual participants” to state that individuals with any symptoms of acute upper respiratory tract infection were excluded at the time of inoculation, to minimise the risk of secondary pneumococcal disease.***
